# Causal Forests Versus Inverse Probability of Treatment Weighting to Adjust for Cluster‐Level Confounding: A Parametric and Plasmode Simulation Study Based on US Hospital Electronic Health Record Data

**DOI:** 10.1002/pds.70257

**Published:** 2025-11-03

**Authors:** Mike Du, Stephen Johnston, Paul M. Coplan, Victoria Y. Strauss, Sara Khalid, Daniel Prieto‐Alhambra

**Affiliations:** ^1^ Pharmaco‐ and Device Epidemiology Group, Health Data Sciences, Botnar Research Centre, NDORMS University of Oxford Oxford UK; ^2^ Epidemiology & Real‐World Data Sciences, MedTech, Johnson & Johnson New Brunswick New Jersey USA; ^3^ Boehringer Ingelheim Pharma GmbH and Co KG Ingelheim Rheinland‐Pfalz Germany; ^4^ Department of Medical Informatics Erasmus Medical Center University Rotterdam the Netherlands

**Keywords:** causal forests, causal inference, clustered data, machine learning, propensity score, simulation study

## Abstract

**Background:**

Rapid innovation and new regulations increase the need for post‐marketing surveillance of implantable devices. However, complex multi‐level confounding related to patient‐level and surgeon or hospital covariates hampers observational studies of risks and benefits. We conducted two simulation studies to compare the performance of Causal Forests (CF) versus Inverse Probability of Treatment Weighting (IPTW) to reduce confounding bias in the presence of strong surgeon impact on treatment allocation.

**Methods:**

Two Monte Carlo simulation studies were carried out: (1) Parametric simulations with patients nested in clusters (ratio 10:1, 50:1, 100:1, 200:1, 500:1) and sample size *n* = 10 000 were conducted with patient and cluster level confounders; (2) Plasmode simulations generated from a cohort of 9981 patients admitted for pancreatectomy between 2015 and 2019 from the US PINC AT hospital research database. Different CF algorithms and IPTW were used to estimate binary treatment effects.

**Results:**

Performance varied with the strength of cluster‐level confounding. Under weak to moderate surgeon influence, CF and IPTW performed similarly. When confounding was strong (OR = 2.5), CF reduced bias compared with IPTW: in parametric simulations, relative bias averaged 11.2% for CF versus 19.9% for IPTW, with similar advantages observed in plasmode simulations.

**Conclusions:**

CF shows promise as a method for estimating treatment effects in scenarios where cluster‐level confounding strongly impacts treatment allocation. More research is needed to guide its use.

**Summary**

- Causal Forests using the double regression tree algorithm showed the least bias in scenarios with strong cluster‐level confounding and small cluster size.
- Including the cluster‐ID indicator in Causal Forests resulted in higher bias and empirical standard error in scenarios with fewer but larger clusters.
- Simulation evidence shows that Causal Forests performed slightly better than IPTW in scenarios with strong cluster‐level confounding.

## 1 Introduction

Observational studies using routinely collected patient data, such as data from health registries, hospitals or insurers, have become essential for clinical treatment comparative studies, especially when randomised controlled trials (RCTs) are unfeasible or unethical [1]. In surgical and medical device epidemiology, treatment allocation is not only influenced by patient characteristics but also by hospital and physician/surgeon characteristics, such as surgeon expertise and hospital policies, leading to clustered data. These extra cluster covariates can introduce confounding by indication that is more complex than general pharmacoepidemiology studies [2]. Hence, failing to adjust for these cluster‐level covariates could affect the accuracy of the treatment effect estimates due to confounding bias from the cluster‐level covariates.

Rosenbaum and Rubin [3, 4] introduced the propensity score (PS) to adjust for confounding, and while PS‐based methods are popular in pharmacoepidemiology, they were initially designed without taking the clustered nature of data [5]. However, recent literature has extended PS methods to accommodate clustered settings and offered strategies to adjust for cluster‐level confounding, such as the inclusion of clustered covariates and the use of fixed or random effects models in PS‐based methods [6, 7, 8]. Despite these refinements, PS‐based methods still rely on correctly specified models and can be sensitive to misspecification, particularly in more complex settings like surgical and medical device epidemiology, where treatment decisions are often influenced by surgeon or hospital‐level covariates [9, 10].

Recent advancements in machine learning techniques offer alternative solutions to these challenges, and the performance has been evaluated against clustered data in several recent studies [11, 12, 13, 14]. Among these, Causal Forests, a random Forest‐based algorithm, has emerged as a promising method for estimating treatment effects in observational studies [15]. The Causal Forest algorithm modifies the traditional Random Forest algorithm [16] for treatment effect estimation.

Causal Forests, a machine learning method primarily developed to estimate heterogeneous treatment effects, are based on decision trees known as Causal Trees, first proposed by Wager et al. [12, 13]. Similar to PS‐based methods, Causal Forests are also built upon the potential outcomes framework and rely on the assumption of no unmeasured confounding [3]. Although the algorithm was initially proposed to estimate individual‐level treatment effects, the average treatment effect (ATE) can be obtained by averaging these conditional estimates across the population.

The first step in the Causal Forests algorithm is the construction of an adjusted outcome for each observation in the sample. The adjusted outcome is usually estimated using a doubly robust estimator that uses a propensity score model and outcome models [5, 17]. The formulae for the construction of the doubly robust estimator used for Causal Forest can be found in the works of Wager et al. [12] and Susan et al. [13]. Hence, these adjusted outcomes are adjusted for confounding and can serve as the learning targets for the Causal Forest.

Then the algorithm begins by dividing the full sample into two subsamples and applying bootstrap aggregation [16]. Each subsample is then split into two parts: one for determining tree splits and the other for estimating treatment effects. This double regression splitting reduces bias by separating the tree split determination from treatment effect estimation from the adjusted outcome estimated from the doubly robust estimator, though it may result in lower precision due to reduced data usage. The trees continue splitting until further divisions no longer improve the heterogeneity of treatment effects or predefined criteria, such as minimum leaf size or maximum depth, are met. The final ATE is calculated as a weighted average of the effects from each tree [12].

While several studies have compared Causal Forests with propensity score‐based methods and demonstrated that Causal Forests show accuracy similar to those of propensity score methods [15, 18], limited attention has been given to the performance of Causal Forests in surgical epidemiology settings. In these settings, cluster‐level covariates may significantly impact treatment allocation, potentially influencing the accuracy of treatment effect estimates. Therefore, we aimed to compare the precision and bias of Causal Forests with Inverse Probability of Treatment Weighting (IPTW), a method that has been shown to be less accurate in scenarios with strong cluster‐level confounders' impact on treatment allocation from a previous study we conducted [19]. Our study focuses on various scenarios with differing degrees of cluster‐level confounding on treatment allocation. To evaluate this, we employed parametric and plasmode simulations [20, 21], in which covariates were simulated based on a real‐world dataset.

## 2 Methods

### 2.1 Parametric Simulation Data Generation Process

The simulation settings were based on previous simulation studies with clustered data [7, 22], but with parameters chosen to mimic the structure of a real‐world dataset described below. We simulated the datasets via parametric simulations [23, 24, 25] with a fixed sample size of 10 000 individuals to represent the patients, binary treatment allocation (*T*) and binary outcome (*Y*). The datasets contain seven patient‐level covariates (*x*1–*x*7), two cluster‐level covariates (*z*1 and *z*2 to represent potential hospital‐level or surgeon‐level confounders), and a cross‐level interaction term between the individual and cluster‐level confounders, which were simulated for each patient. Among the individual covariates simulated, five were confounders (*x*1–*x*5), one (*x*6) was an instrumental variable associated with the treatment but not with the outcome (other than through the treatment) and *x*7 was a risk factor associated with the outcome but not the treatment [26]. Both cluster‐level covariates (*z*1 and *z*2) were generated as confounders associated with treatment and outcome. The cluster and patient‐level covariates were simulated from different probability distributions to reflect different covariates observed in real‐world medical devices or surgical data.

**FIGURE 1:**
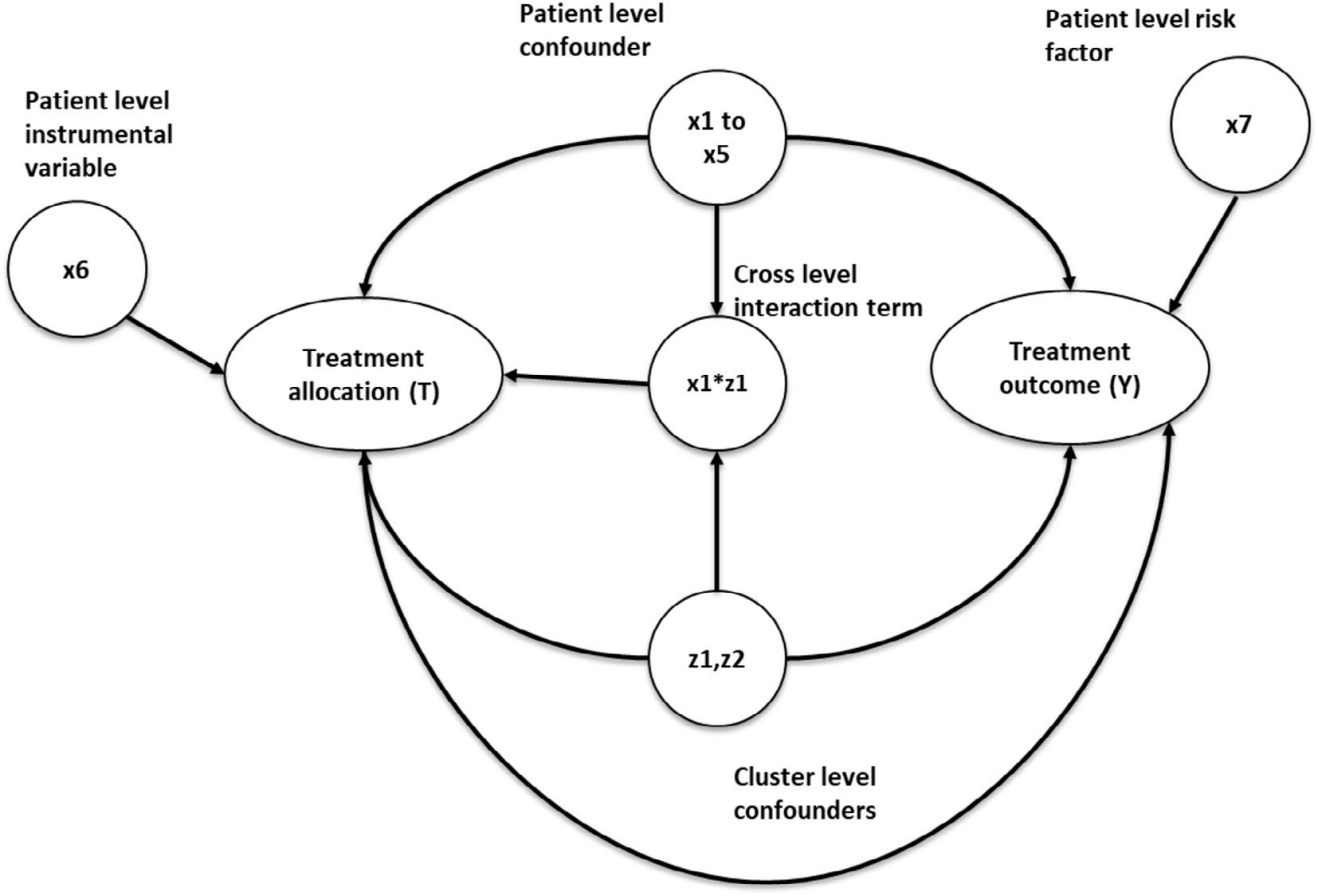
This diagram gives the causal relationship between the covariates in the simulation data; the arrow indicates causes. For example, *x*1 → *Y* implies *x*1 causes *Y*.

Twenty different scenarios were simulated to test the performance of the proposed methods for controlling cluster‐level confounding. The scenarios were generated by varying the cluster structure of the data and the effect size of the cluster‐level confounders on treatment allocation (*z*1 and *z*2), ranging from negligible with odds ratio = 1.01 to odds ratio = 2.5 to resemble strong cluster‐level confounding. Five different cluster structures were simulated with different cluster numbers (*m*) and average patients per cluster (*n*) (*m* = 10, *n* = 1000), (*m* = 50, *n* = 200), (*m* = 100, *n* = 100), (*m* = 200, *n* = 50) and (*m* = 500, *n* = 20). Patients per cluster (*n*) were randomly sampled from the Poisson distribution with mean *n* for each cluster within the dataset. Table 1 gives the 20 different simulation data scenarios generated. Figure 1 gives the causal diagram of the simulation covariates and the simulations are run for 100 repetitions.

**TABLE 1:** The table gives the generation distribution, effects on treatment allocation and effects on treatment outcome for covariates generated in the simulations.

Covariates	Description	Effects on treatment allocation	Effects on treatment outcome	Generation distribution
Cluster structure (*m*, *n*)	*m* = number of cluster in the data *n* = average number of patients per a cluster	N/A	N/A	*m* = fixed number with 10, 50, 100, 200, 500. *n* = poisson (1000, 200, 100, 50, 20)
*z*1, *z*2	Cluster‐level confounders	*z*1 = *z*2 = [0.01, 0.2231, 0.4055, 0.9163] ~ [OR = 1.01, 1.25, 1.5, 2.5]	*z*1 = *z*2 = 0.4055 (OR = 1.5)	*z*1 = normal (0, 1), *z*2 = Bernoulli (0.5)
*x*1–*x*5	Individual level confounders	[*x*1, *x*2, *x*3, *x*4] = [0.35, 0.4, 0.45, 0.55]	[*x*1, *x*2, *x*3, *x*4] = [0.35, 0.4, 0.45, 0.55]	[*x*1, *x*2, *x*3] = Bernoulli ([0.4, 0.45, 0.5]) *x*4, *x*5 = normal (0, 1)
*x*6	Individual level risk factor	0	0.5	Bernoulli (0.5)
*x*7	Individual level instrumental variable	0.5	0	Bernoulli (0.5)
*z*1*x1	Cross level interaction term	0.4055 (OR = 1.5)	0	*z*1*x1
*T*	True treatment effect	N/A	0.4055 (OR = 1.5)	N/A

Abbreviation: OR, Odds ratio.

### 2.2 Plasmode Simulation Data Generation Process

Plasmode simulation [21, 27] is a method that generates synthetic data by re‐sampling from pre‐selected observed covariates of a real‐world dataset. The re‐sampling of covariates was performed using the bootstrap with replacement method [28]. The exposure and outcome of Plasmode simulation data are generated using the investigators' pre‐specified re‐sampled covariates from the real‐world data cohort and choice of true treatment effects. Hence, simulated data generated using Plasmode simulation will preserve the data structure and covariates of the real‐world data cohort from which it was generated. The key advantage of plasmode simulation is preserving realistic complexity in terms of the relationship between covariates. However, Plasmode simulation lacks the ability to change its data structure.

The real‐world data cohort we generated in our Plasmode simulation was from the US PINC AI Healthcare Database, an all‐payer hospital database collected from among over 1000 hospitals in the US [29]. The Premier Healthcare Database includes information from hospitals' electronic health records, including diagnoses, procedures, patient characteristics and hospital features. The cohort included 9981 patients aged 18 or over who were admitted for pancreatectomy from 2015 to 2019. The patients' covariates of the simulated data were generated based on age, sex and the Charlson comorbidity index. The cluster of the simulated data was identified using the hospital ID. The cohort had 341 unique hospital IDs, with an average of 30 patients per hospital. The cluster covariates were re‐sampled from hospital‐related covariates such as type (teaching or not teaching), hospital size (500+ or 500 fewer beds) and the hospital's yearly pancreatectomy volumes, and so forth. Same as the parametric simulation, the true treatment effect was set at OR = 1.5 in all the scenarios. A full summary of the covariates used in the Plasmode simulation is provided in the Figures S1 and S2.

### 2.3 Comparison of IPTW and Causal Forests

Both IPTW and CF can be used to estimate an ATE. IPTW directly reweights individuals based on the inverse of their estimated propensity scores to achieve covariate balance between treatment groups, thereby targeting a marginal ATE. In contrast, CF estimates conditional average treatment effects (CATE) at the individual level and then averages them to yield an overall ATE. Hence, these two approaches are comparable for the estimation of ATE in the simulation study proposed. Table 2 summarises the key differences between IPTW and CF.

**TABLE 2:** Comparison of Inverse Probability of Treatment Weighting (IPTW) and Causal Forest (CF) approaches for estimating the average treatment effect (ATE), including key components and typical implementations.

Component	Inverse Probability of Treatment Weighting	Causal Forest
Primary purpose and estimand	Adjust for confounding to estimate average treatment effects.	Estimate heterogeneous treatment effects for subgroups internal to the causal trees; however, they can be averaged to estimate the average treatment effects.
Estimation approach	Inverse probability weighting using propensity scores	Doubly robust estimation combining propensity scores and outcome models using regression forests
Propensity score estimation	Usually Logistics regression	Usually by a regression forest, but an external propensity scores model can also be used
Outcome	Not required	Regression forests for outcome estimation
Main strength	Efficient if the propensity score model is correctly specified	Flexible, less sensitive to extreme weights
Main limitation	Unstable with extreme weights and model‐dependent	Less transparent, requires large sample sizes for stable estimation.

### 2.4 Implementation of IPTW

For IPTW, the propensity scores were estimated using a logistic regression propensity score model with cluster‐level and patient‐level confounders as covariates. Then, the ATEs were estimated using a random effects model. The treatment outcome was regressed on the treatment allocation using a random intercept logit model weighted with stabilised IPTW calculated from the PS model. The method used is similar to the IPTW methods used in previous methodology research for clustered data, which have been shown to be effective in reducing bias due to cluster‐level confounding [30].

### 2.5 Implementation of Causal Forests

Three different Causal Forest approaches were implemented using the grf package in R (version 2.2.0) [31] to assess the treatment effect estimations of the two different simulation studies. (1) CF—Using the standard ‘causal_forest()’ function, without explicitly adjusting for clustering. Treatment effects were adjusted using doubly robust pseudo‐outcomes with propensity score and outcome model built with the internal regression tree model from the grf package. (2) CF–clusterID—Implemented the same as (1) but with the ‘clusters’ argument inside ‘causal_forest()’ specified as the cluster label in the simulated dataset. This meant the causal tree is built using within‐cluster sampling; hence observations from the same cluster are either included or excluded together when growing the Causal tree to ensure cluster robust inference. (3) CF–PS—Same as (1) but with an externally estimated propensity score to adjust the treatment effects for the causal forest. The same propensity score model was used as the one used for IPTW, which was built using a logistic regression model that included both the cluster and patient‐level confounders as covariates. This is achieved via the ‘W.hat’ argument in the ‘causal_forest()’ function. This configuration allows direct comparison of the Causal Forest with the IPTW approach using the same propensity score model. Other default settings of the grf package were used for other tuning parameters and were kept constant for all three different Causal Forest approaches. The major tuning parameter for the default settings is the number of trees grown in the forests, which was set to 200; the number of forests used was set to 50, and the fraction of data that was used for determining the splits was set to 50%. More details can be found on the package website for the grf package [31]. Table 3 also provides a summary of all the causal inference methods implemented in this study.

**TABLE 3:** Summary of the causal inference methods compared in this study.

Method
(PS–IPTW)—Inverse Probability of Treatment Weighting using propensity scores estimated via logistic regression. The model includes both patient‐level and cluster‐level confounders as covariates.
(CF)–Causal Forests using the default causal forest implementation with no explicit clustering adjustment. Treatment effects are adjusted using doubly robust estimator, with both the propensity score and outcome models estimated internally using regression forests.
(CF–clusterID)—Causal Forests implemented as in CF, but with the clusters argument in the ‘grf’ package set to the cluster ID of the simulated data. This ensures cluster‐sampling and cluster‐robust inference by including or excluding entire clusters during the construction of the causal trees.
(CF–PS)—Causal Forests using the same setup as CF, but with externally estimated propensity scores provided via the ‘W.hat’ argument of ‘grf’ package. These scores are estimated from the same propensity score model used in the PS–IPTW method.

### 2.6 Assessment of Results

The precision and accuracy of the estimated treatment effects for both propensity score weighting and Causal Forests were compared. Using average absolute relative bias (Rbias), empirical standard error (EmpSE) and 95% confidence intervals model coverage (95% Coverage) of the 1000 repetitions for each simulated scenario as defined in the guidance literature on simulation studies by Morris et al. [32]. All analyses were performed in R version 4.3.1, with the Parametric simulation data generated with the ‘simstudy’ package [33] and the Plasmode simulation data generated with the ‘Plasmode’ package [21].

## 3 Results

**FIGURE 2:**
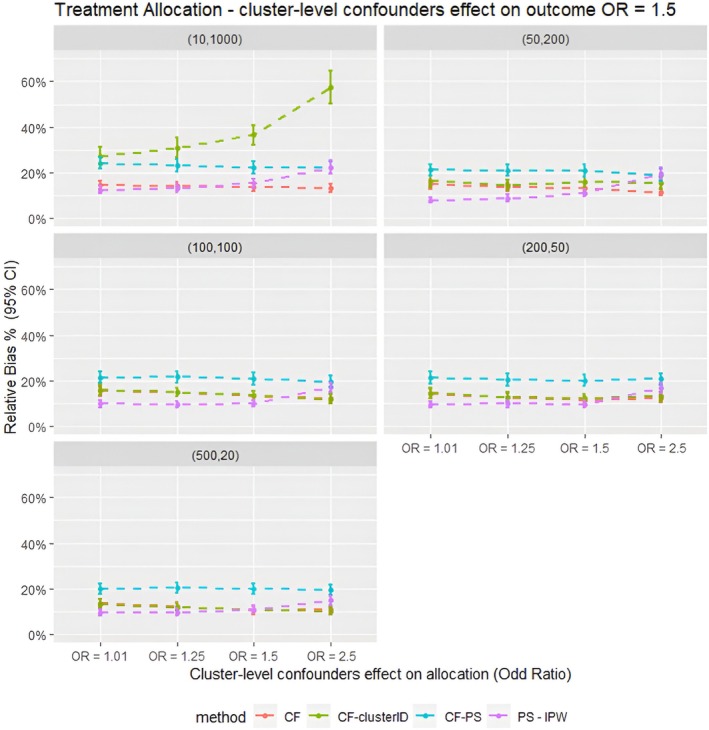
Average relative bias for different confounder effects on treatment allocation scenarios for different methods for all the data scenarios tested in the parametric simulation study. PS–IPTW, Inverse Probability of Treatment Weighting using propensity scores method, explained in the method section; CF, Causal Forests using double tree method; CF–clusterID, Causal Forests using double tree methods with the cluster label included as input for the Causal Forests; CF–PS, Causal Forests using propensity score as decision parameters for splitting. (XX,XX) = cluster structure with (number of clusters, average patients per cluster).

**FIGURE 3:**
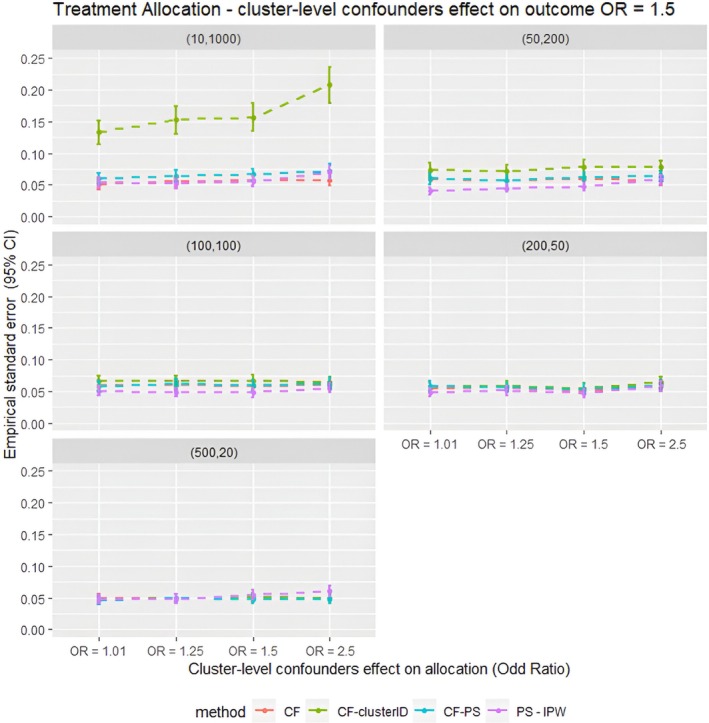
Average empirical standard error for different confounder effects on treatment allocation scenarios for different methods for all the data scenarios tested in the parametric simulation study. PS–IPTW, Inverse Probability of Treatment Weighting using propensity scores method, explained in the method section; CF, Causal Forests using double tree method; CF–clusterID, Causal Forests using double tree methods with the cluster label included as input for the Causal Forests; CF–PS, Causal Forests using propensity score as decision parameters for splitting. (XX,XX) = cluster structure with (number of clusters, average patients per cluster).

**FIGURE 4:**
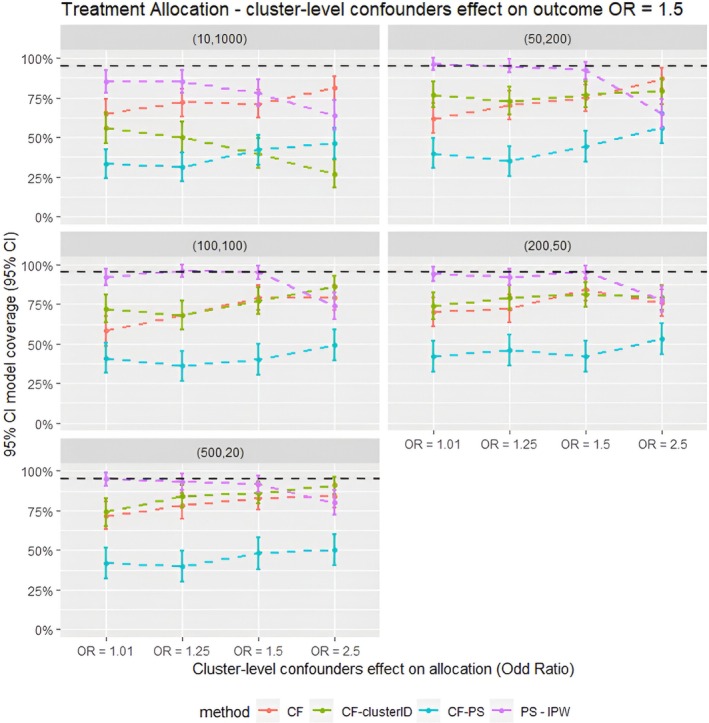
Average model coverage for different confounder effects on treatment allocation scenarios for different methods for all the data scenarios tested in the parametric simulation study. PS–IPTW, Inverse Probability of Treatment Weighting using propensity scores method, explained in the method section; CF, Causal Forests using the double tree method; CF–clusterID, Causal Forests using double tree methods with the cluster label included as input for the Causal Forests; CF–PS—Causal Forests using propensity score as decision parameters for splitting. (XX,XX) = cluster structure with (number of clusters, average patients per cluster).

**FIGURE 5:**
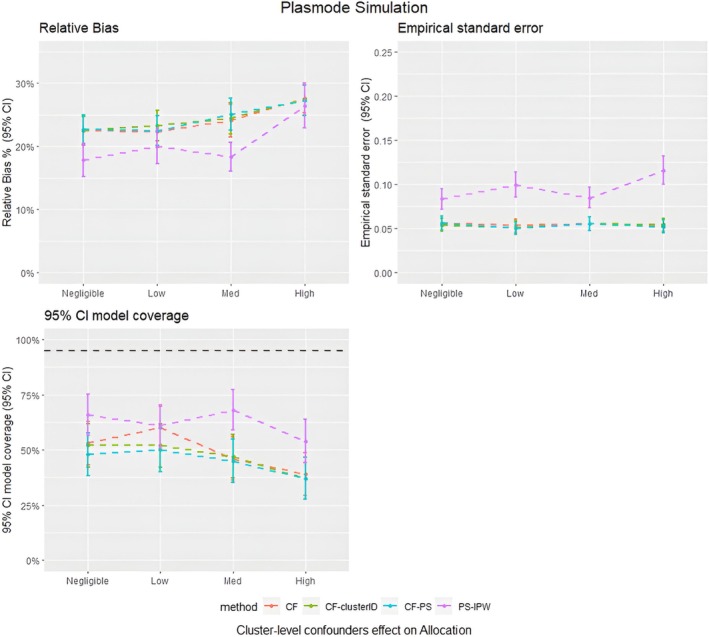
Average relative bias, model coverage and empirical standard error for different matching strategies for all the data scenarios tested in the Plasmode simulation study. PS–IPTW, Inverse Probability of Treatment Weighting using propensity scores method, explained in the method section; CF, Causal Forests using double tree method; CF–clusterID, Causal Forests using double tree methods with the cluster label included as input for the Causal Forests; CF–PS, Causal Forests using propensity score as decision parameters for splitting.

The findings from the parametric simulations, shown in Figures 2, 3, 4, 5, indicate that CF using the double regression tree approach performed the best out of the three different methods tested in this study. CF gave the lowest relative bias and empirical standard error compared to CF–PS. For example, in the cluster structure scenario with *m* = 10 and *n* = 1000, with cluster‐level confounders effect on treatment allocation, OR = 1.5, CF had a relative bias of 13.8%. In comparison, CF–PS had a relative bias of 22.2%. These results suggest that changing the propensity scores model for treatment adjustment within the Causal Forest did not improve the accuracy and precision of the treatment estimate for the clustered data scenarios tested in this simulation study.

Moreover, including the cluster ID indicator as input for the Causal Forest (CF–clusterID) did not improve the accuracy of CF. Instead, it substantially decreased the accuracy of CF in the cluster structure scenarios with *m* = 10 and *n* = 1000. For example, the relative bias for CF was 14.0%, compared with 30.9% for CF–clusterID in the cluster‐level confounder effects on treatment allocation (OR = 1.25) scenario. However, as the number of clusters in the structure increased and the number of individuals per cluster decreased, the differences in relative bias between CF and CF–clusterID also decreased. Nevertheless, CF still had slightly lower relative bias and empirical standard error in the scenarios tested. These findings suggest that including the cluster ID as input for Causal Forest is unnecessary for data with cluster‐level confounding, regardless of the cluster structure of the data.

Additionally, when comparing CF with IPTW, the parametric simulation results indicated that IPTW generally outperformed CF in most scenarios. For instance, in scenarios with a cluster structure of *m* = 50 and *n* = 200 and cluster‐level confounding on treatment allocation (OR = 1.25), the relative bias was 8.93% for IPTW compared to 13.7% for CF. However, CF provided more accurate estimates when the cluster‐level confounding effect was strong (OR = 2.5). For example, in scenarios with a cluster structure of *m* = 500 and *n* = 20 and cluster‐level confounding effect on allocation of OR = 2.5, the relative bias was 11.1% for CF compared with 19.7% for IPTW. These results suggest that CF could be a suitable alternative to IPTW in scenarios with strong cluster‐level confounders' effect on treatment allocation because it gives results with lower bias under such conditions.

Furthermore, as shown in Figure 3, the parametric simulations showed that the empirical standard error was similar for all the methods compared in this study, except for CF–clusterID, which had substantially higher standard error in scenarios with *m* = 10 and *n* = 1000. This suggests that the precision of Causal Forest‐based methods was comparable to that of IPTW. Consequently, the trends observed in terms of 95% CI model coverage (Figure 4) were similar to those observed for the relative bias. In scenarios with a strong cluster‐level confounding effect on allocation (OR = 2.5), CF offered higher model coverage. Conversely, in other scenarios, IPTW gave higher model coverage than CF.

The Plasmode simulation results (Figure 5) also show that the performance of the CF and PS–IPTW methods in estimating treatment effects is comparable to those of the parametric simulation, which had a cluster structure with *m* = 500 and *n* = 20. Specifically, the results for CF and CF–clusterID were almost identical, indicating that adding the cluster ID indicator sample splitting does not affect treatment effect estimates in data with a large number of clusters and small cluster sizes. The PS–IPTW method showed slightly better accuracy in treatment estimates compared to CF, with lower relative bias, regardless of the strength of the cluster effect on treatment allocation. However, the 95% confidence intervals of the relative bias for both methods overlapped, and the model coverage of the Plasmode simulation was also similar between the two methods. This suggests that the improvement in accuracy of PS–IPTW over CF was minimal, consistent with the findings from the parametric simulation.

However, there were notable differences between the plasmode and parametric simulation results. First, CF–PS performed similarly to CF in the plasmode simulation, whereas in the parametric simulation it showed higher bias and lower coverage. Second, the empirical standard error for PS–IPTW was higher than that for CF methods in the plasmode setting, while in the parametric simulation they were similar. Third, in the plasmode simulations, CF's bias increased as the cluster‐level confounder effect on treatment allocation increased, a trend not observed in the parametric simulations. These differences likely reflect the higher complexity and potential model misspecification in the plasmode data, which contained more covariates at both the individual and cluster levels and a different cluster structure.

## 4 Discussion

This study reveals several important findings regarding the performance of Causal Forests compared to IPTW. In general, IPTW tended to provide more accurate treatment effect estimates than CF in most scenarios tested, particularly in the parametric simulations with weak to moderate cluster‐level confounding and correctly specified models. This is expected and consistent with current literature, as IPTW is expected to be efficient when its propensity score model is correctly specified [3, 7]. However, we found that when cluster‐level confounding effects on treatment allocation were strong, CF sometimes offered more accurate treatment effect estimates than IPTW. Specifically, in scenarios with strong cluster‐level confounding effects, the bias for IPTW tended to increase, while the bias for CF did not increase as much, particularly in the parametric simulation. This is likely due to the presence of extreme propensity score weights for IPTW from the strong cluster‐level confounders. As previous literature has shown, this is a common problem for IPTW and can cause bias in the average treatment estimates [34, 35]. Causal Forests, by contrast, do not rely on IPTW in its estimation process, and the results are more robust in those scenarios with strong cluster‐level confounders in the simulation study.

The comparisons between different Causal Forest methods showed that incorporating the cluster‐ID indicator for within‐cluster sampling in Causal Forests did not have a noticeable effect on the accuracy or precision of the treatment effects in the majority of the simulation settings tested. However, in situations where the number of clusters was small and the cluster size was large, the addition of the cluster‐ID indicator resulted in significantly higher bias and empirical standard error when compared to Causal Forest methods without incorporating the cluster‐ID indicator. Furthermore, providing externally estimated propensity scores from the same logistic regression model used in IPTW to the CF doubly robust estimator (CF–PS) did not improve performance compared with CF using internally estimated propensity scores from regression forests. These findings align with those reported in a prior simulation study conducted by Suk et al. [15]. Hence according to the findings of this simulation study, Causal Forest using the double regression tree method without the inclusion of the cluster‐ID indicator is the most effective of the three Causal Forest models for the estimation of treatment effect for clustered data with confounders from cluster‐level.

We found some discrepancies in the findings between the Plasmode and parametric simulations. This could be caused by the differences in the data used for the simulation. For instance, the Plasmode simulations included a larger number of covariates at both the individual and cluster levels, while the cluster structure of the data was different between the two simulations. Furthermore, due to the real‐world nature of the plasmode simulation, the data would be more complex than the parametric simulation; for example, the covariates would likely be correlated and contain non‐linear relationships between them. Hence, this could be the reason the majority of the results have overlapping 95% confidence intervals, hence suggesting similar performance. Given these differences in the data, it is difficult to determine the exact causes of the differences in the results between the Plasmode and parametric simulations without further investigation with extra scenarios simulated.

## 5 Strengths and Limitations

This study's main strength is its use of simulated data, where the actual treatment effect was known. Hence using simulation studies allowed us to calculate the bias and empirical standard error for the treatment effects estimated using different methods. Therefore, the accuracy and precision of different methods can be compared. Using simulated data also allowed us to create different scenarios by varying data variables to see how each PS method behaves in different scenarios. This is usually difficult to achieve in real‐world data analysis.

This study was also subject to limitations. A major limitation of this study is that it has not captured all cluster structure, cluster‐confounding effect sizes and covariate scenarios that may occur in real‐world data. Hence, the findings may only be generalisable to the scenarios tested in this simulation study. Besides the limitation of the simulation setting, this study did not evaluate the tuning parameters for Causal Forests such as the proportions of samples used for the split in the double regression tree algorithm, tree depths and different stopping criteria, as currently there is limited guidance on tuning Causal Forests in epidemiology research [18]. It is fair to argue that better performance can be achieved by experimenting with different tuning parameters for Causal Forests.

## 6 Conclusion

This study provides valuable insights into the use of Causal Forests for estimating ATEs in clustered data. The results demonstrated that the performance of Causal Forests was comparable to IPTW, with Causal Forests showing higher accuracy in scenarios where cluster‐level confounders strongly influenced treatment allocation. This suggests that Causal Forests could potentially serve as an alternative method for analysing ATEs in medical device and surgical epidemiology, particularly in scenarios where surgeons have a significant impact on treatment allocation.

However, further empirical research is needed to offer more guidance and insight on the use of Causal Forest in real‐world studies. Prior research on the application of Causal Forest was more focused on its ability to estimate heterogeneous treatment effects between subgroups and research on estimating ATEs in clustered data is limited. It would be useful to investigate how Causal Forest behaves in other sample sizes, cluster‐confounding effects on outcomes, its ability to handle unmeasured confounders, missing data and interactions.

In conclusion, while Causal Forests show promise as a method for estimating ATEs based on observational clustered data, further research is needed to understand their strengths and limitations fully. The results of this study provide a useful starting point and motivation for future research into the applications of Causal Forest methods for causal inference in observational studies of surgical epidemiology and medical device research.

### 6.1 Plain Language Summary

This study compared two methods for estimating treatment effects in clustered observational health data where the healthcare provider, such as a hospital or surgeon, influences patient outcomes and treatment allocation. Using simulated data, we evaluated a machine learning method called Causal Forest and compared it to a commonly used approach called IPTW. We found that Causal Forest, particularly when using the double regression tree technique, performed well in many scenarios and gave less bias when provider‐related factors strongly influenced the treatment allocation. While IPTW generally produces accurate results, it depends on strong assumptions that may not hold in real‐world studies. Our findings suggest that Causal Forest is a promising approach for estimating treatment effects in clustered observational health data settings, particularly in surgical and medical device research where treatment decisions vary by provider.

## Author Contributions

D.P.‐A. and M.D. led the conceptualisation of the study with contributions from S.J., S.K., V.Y.S., P.M.C. and S.J. M.D. developed the code for statistical analyses and simulations. M.D. wrote the first draft of the manuscript. All authors read, contributed to, and approved the last version of the manuscript.

## Ethics Statement

The authors have nothing to report.

## Consent

The authors have nothing to report.

## Conflicts of Interest

D.P.‐A.'s research group from the University of Oxford has received research grants from the Innovative Medicines Initiative, from Gilead Science, from Theramex, and from UCB Biopharma, none of which are related to this manuscript. S.J. and P.M.C. are employees of Johnson & Johnson Medical Device Companies and the Office of the Chief Medical Officer. V.Y.S. is an employee at Boehringer‐Ingelheim Pharma GmbH & Co., KG.

## Supporting information


**Data S1:** pds70257‐sup‐0001‐Supinfo.docx.

## Data Availability

The study code for the study is available on GitHub. Please email mike.du@ndorms.ox.ac.uk for access.
